# Isolation and Characterisation of Mesenchymal Stem/Stromal Cells in the Ovine Endometrium

**DOI:** 10.1371/journal.pone.0127531

**Published:** 2015-05-18

**Authors:** Vincent Letouzey, Ker Sin Tan, James A. Deane, Daniela Ulrich, Shanti Gurung, Y. Rue Ong, Caroline E. Gargett

**Affiliations:** 1 The Ritchie Centre, MIMR-PHI Institute of Medical Research, Clayton, Victoria Australia; 2 Department of Obstetrics and Gynaecology, Monash University, Clayton, Victoria Australia; National Institutes of Health, UNITED STATES

## Abstract

**Objective:**

Mesenchymal stem/stromal cells (MSC) were recently discovered in the human endometrium. These cells possess key stem cell properties and show promising results in small animal models when used for preclinical tissue engineering studies. A small number of surface markers have been identified that enrich for MSC from bone marrow and human endometrium, including the Sushi Domain-containing 2 (SUSD2; W5C5) and CD271 markers. In preparation for developing a large animal preclinical model for urological and gynecological tissue engineering applications we aimed to identify and characterise MSC in ovine endometrium and determine surface markers to enable their prospective isolation.

**Materials and Methods:**

Ovine endometrium was obtained from hysterectomised ewes following progesterone synchronisation, dissociated into single cell suspensions and tested for MSC surface markers and key stem cell properties. Purified stromal cells were obtained by flow cytometry sorting with CD49f and CD45 to remove epithelial cells and leukocytes respectively, and MSC properties investigated.

**Results:**

There was a small population CD271^+^ stromal cells (4.5 ± 2.3%) in the ovine endometrium. Double labelling with CD271 and CD49f showed that the sorted CD271^+^CD49f^-^ stromal cell population possessed significantly higher cloning efficiency, serial cloning capacity and a qualitative increased ability to differentiate into 4 mesodermal lineages (adipocytic, smooth muscle, chondrocytic and osteoblastic) than CD271^-^CD49f^-^ cells. Immunolabelling studies identified an adventitial perivascular location for ovine endometrial CD271^+^ cells.

**Conclusion:**

This is the first study to characterise MSC in the ovine endometrium and identify a surface marker profile identifying their location and enabling their prospective isolation. This knowledge will allow future preclinical studies with a large animal model that is well established for pelvic organ prolapse research.

## Introduction

Tissue engineering (TE) is the combination of a range of biological and synthetic material scaffolds with a variety of cell types and has revolutionized treatment options for several clinical conditions. TE approaches have for instance been used to generate new tissues and organs [1] including the bladder and vagina [2], and to improve long-term outcomes of surgical interventions. TE approaches using stem cells and in particular mesenchymal stem/stromal cells (MSC) are most promising because they possess key properties; self-renewal, high proliferative potential and differentiation. However, the main action of MSC whether transplanted with or without material scaffolds appears to be through paracrine action on endogenous cells through their release of numerous factors [3].

Mesenchymal stem cells or mesenchymal stromal cells (MSC), originally identified in the bone marrow are defined as plastic adherent cells with a characteristic surface phenotype, colony-forming ability, and multipotency by differentiating *in vitro* into adipogenic, chondrogenic and osteogenic mesodermally-derived lineages [4]. More recently, MSC have been identified in most human tissues including umbilical cord blood, adipose tissue and endometrium [5–8]. Human endometrium contains a small population of clonogenic stromal cells with typical MSC properties [9–11]. Endometrial MSC (eMSC) have also been identified as a component of endometrial side-population (SP) cells [11–14]. The eMSC are clonogenic and self-renew as demonstrated by serial cloning in culture [10]; they undergo multilineage differentiation into four mesenchymal lineages, including smooth muscle cells *in vitro*, indicating their similarity to bone marrow MSC. Endometrial MSC also generate endometrial stroma in xenograft assays [14, 15]. One advantage of human eMSC is the relative ease with which they can be obtained by an endometrial biopsy as an office-based procedure without the use of anaesthesia, which is significantly less painful or invasive than bone marrow aspiration or liposuction [16].

MSC have typically been obtained from bone marrow and menstrual blood by adherence to plastic cultureware [4, 16] and from culture of the stromal vascular fraction of unfractionated adipose tissue [17]. These cultures are heterogeneous, comprising stromal, endothelial and perivascular cells and are not particularly enriched for MSC, although a small percentage MSC will be present. The advantage of using specific surface markers for prospective isolation of MSC is that enriched populations are obtained which contain the majority of the clonogenic, self-renewing cells that will produce many more cells upon culture expansion [5]. Several single cell surface markers, distinct from the commonly expressed MSC phenotypic markers, have been used to enrich MSC from various tissue sources, including Stro-1 [18], CD271 also known as NGFR (low-affinity nerve growth factor receptor) or p75 NTR (neurotrophin receptor) [19] and MSCA-1 (Tissue Non-specific Alkaline Phosphatase, TNAP) [20, 21] Ovine bone marrow MSC (bmMSC) have been prospectively isolated from bone marrow and adipose tissue using Stro-4 [22].

Specific markers have now been identified that enrich for human eMSC [7, 15]. Almost all clonogenic stromal cells with MSC properties are found in the CD146^+^PDGFRB^+^ and W5C5^+^ subpopulations of endometrial stromal cells. The epitope for the W5C5 antibody is Sushi Domain-containing 2 (SUSD2) [23]. These markers revealed that eMSC are located around blood vessels as pericytes in both the functionalis and basalis layer of human endometrium. They also enable the prospective isolation of eMSC from biopsy, curettage or hysterectomy tissue.

The aim of this study was to determine if MSC were present in ovine endometrium, develop methods for isolating MSC from ovine endometrium and characterize their adult stem cell properties of self-renewal, high proliferative potential and differentiation. This is an important step forward in the strategy for developing an autologous preclinical large animal model to assess new generations of TE constructs comprising eMSC and novel materials.

## Materials and Methods

### Animal Welfare

The animal surgery was done at the Monash University Melbourne, with approval from the Monash Medical Centre Animal Ethics Committee A (Ethics no. MMCA 2013/38) for animal experimentation. All experiments were conducted in strict accordance with good practice for the use of animals as detailed in the National Health and Medical Research Council Guidelines in compliance with the Animal Welfare Act. All animals were humanely euthanized according to the current guidelines by intravenous administration with Pentobarbitone sodium into the jugular vein (150mg/kg).

### Ovine Uterine Tissues

Ovine tissues (n = 15) were collected from 3–5 year old parous ewes treated with a progesterone sponge-controlled internal drug release dispenser (CIDR, 30 mg Flugestone acetate, EAZI-BREED) inserted vaginally for two weeks and removed 48 hr prior to tissue collection to initiate estrous. The sheep were placed in dorsal recumbency; a 10 cm lower abdominal midline incision was performed and the uterus exposed. The uterus was removed from the intra-abdominal cavity by ligating the ligaments and then put into ice-cold transport medium (HEPES-buffered DMEM/F-12 medium (Invitrogen) supplemented with 1% antibiotic antimycotic (Invitrogen) [21], stored at 4°C and processed within 18 hours. The uterus was opened via a longitudinal incision along the length of both horns and endometrial tissues were taken from different regions of the uterus both at the caruncular and intercaruncular areas.

### Ovine Endometrial Stromal Cell Isolation

Ovine endometrial tissues dissected from the opened uterus were cut into small pieces and minced using a tissue chopper (MacIllwain tissue chopper; Campden Instruments, Loughborough). Minced tissues were dissociated in 5 mg/ml collagenase type I (Worthington Biochemical Corporation), 40 μg/ml deoxyribonuclease type I (Worthington Biochemical Corporation) and 5 mM glucose in PBS for 1.5 hr at 37°C on a rotator. The enzymatic reaction was terminated by adding an equal volume of DMEM/F-12 medium containing 15 mM HEPES (Life Technologies) supplemented with 5% newborn calf serum (Life Technologies) and 1% antibiotic-antimycotic (Life Technologies)(Bench Medium). Dissociated tissues were then filtered through 70-μm cell strainer (BD Biosciences) and stromal cells were collected in the filtrate. To remove red blood cells, Ficoll-Paque PLUS (GE Healthcare Bio-Sciences AB) was under-layered beneath the cell suspension and centrifuged at 500g for 15 min at room temperature (RT). Cells at the media/Ficoll-Paque interface were collected and washed with Bench Medium.

### Flow Cytometry Sorting

Isolated stromal cells (up to 1 x 10^7^ cells/100 μl) were incubated with antibody combinations of allophycocyanin (APC)-conjugated CD49f (1:10, clone GoH3, rat IgG2a; Miltenyi Biotec) and Alexa Fluor 488-conjugated CD45 (1: 20; mouse IgG1; Life Technologies) or phycoerythrin (PE)-conjugated CD271 (1:10, mouse IgG1; Miltenyi Biotec) and APC-conjugated CD49f in 2% fetal bovine serum/PBS (FBS/PBS) for 30 min on ice in the dark. Cells were then washed and resuspended in 1 μM Sytox Blue to distinguish live and dead cells (Life Technologies) and fluorescence activated cell sorting (FACS) was undertaken on a MoFlow flow cytometer (Beckman Coulter) or an Influx flow cytometer (Becton Dickinson Biosciences).

### Cell Culture and *In Vitro* Colony Forming Assay

Freshly sorted cells were cultured in stromal medium containing DMEM/F-12 (Life Technologies), 10% fetal bovine serum (Life Technologies), 2 mM glutamine (Life Technologies), 0.5 mg/ml primocin, 10 ng/ml basic fibroblast growth factor (FGF2) (Peprotech) used for our studies on human eMSC and incubated at 37°C in 5% CO_2_. Medium was changed every 2–3 days.

For colony forming assays, freshly sorted cells were seeded at very low seeding densities of 10–50 cells/cm^2^ onto fibronectin-coated (10 μg/ml) (BD Biosciences 10cm-dishes (BD Biosciences) and cultured in stromal medium with changes at day 6/7. Fibronectin and FGF2 are included in the medium to assist attachment and establishment of clonal cultures. Colonies were monitored to ensure they were derived from single cells. For subcloning, plates were seeded at the lower density to ensure individual clones were clearly separated to avoid contamination. Clonal cultures were fixed in 10% formalin at day 12 and stained with haematoxylin. Cloning efficiency was determined on plates seeded at the higher density to ensure sufficient clones/plate for statistical purposes. Only colonies with >50 cells were counted and colony forming efficiency was then determined [9].

### Serial Cloning Assay

Several of the largest individual clones on cloning plates containing <30 colonies in total were collected per sample and cell type by trypsinisation in cloning rings and recloned. Cells were counted visually under a phase contrast microscope using an ocular grid and seeded at 5–10 cells/cm^2^ onto fibronectin-coated 10 cm dishes and cultured in stromal medium as above to generate secondary clones. Similarly, several secondary clones were harvested and recloned a second time to generate tertiary clones as previously described [10]. The cloning efficiency at each subcloning was assessed as above.

### *In vitro* Differentiation

To induce adipogenic, osteogenic and myogenic differentiation, sorted ovine endometrial stromal cells (CD271^+^CD49f^-^ and CD271^-^CD49f^-^) were seeded separately at 5,000 to 10,000 cells/cm^2^ on coverslips (Thermo Scientific) in 4-well plates or 24-well plates (BD Biosciences) and cultured in respective differentiation media for 4 weeks as described previously for human cells [10]. For chondrogenic differentiation, 5 x 10^5^ cells were pelleted in 15-mL Falcon tube by centrifugation at 1100 rpm for 5 min at 4°C and cultured in chondrogenic medium to produce a 3D micromass culture [10]. Controls were sorted cells cultured in 1% fetal bovine serum stromal medium. Medium was changed every 2–3 days.

Following 3–4 weeks culture in differentiation or control media, cells on coverslip were fixed and stained with 4% Alizarin Red (pH 4.1), 1% Oil Red O or by immunohistochemistry using an antibody to α-smooth muscle actin (3.6 μg/ml, clone 1A4; Dako) for osteogenic, adipogenic and myogenic differentiation, respectively. Chondrogenic micromass cultures were fixed, processed and paraffin embedded. Sections were stained with 1% alcian blue (Sigma-Aldrich). Stained cells or sections were examined under Olympus BX41 microscope (Olympus), and images taken using Olympus DP25 digital camera (Olympus).

### Flow Cytometric Analysis

FACS sorted CD49f^-^ cells or cultured CD271^+^CD49f^-^ cells were stained with primary antibodies PE-conjugated CD271, PE-conjugated PDGFRB (1.25 μg/ml, mouse IgG1; R&D Systems), PE-conjugated SUSD2, (W5C5 clone, 1:20, mouse IgG1; Biolegend), APC-conjugated CD90 (25 μg/ml, mouse IgG1κ, BD Pharmingen), CD146 (1:2, supernatant, clone CC9, mouse IgG2a; donated by Prof David Haylock, CSIRO, Clayton, Victoria, Australia), CD73 (10 μg/ml, mouse IgG1κ; BD Pharmingen) or CD105 (10 μg/ml, mouse IgG1ƙ; BD Pharmingen). The CD146 samples were incubated with secondary antibody fluorescein isothiocyanate (FITC) conjugated anti-mouse IgG2a (5 μg/ml, clone R19-15; BD Pharmingen). CD73 and CD105 samples were incubated with secondary antibody PE-anti-mouse IgG1 (2 μg/ml, clone A85-1, BD Pharmingen). Isotype matched controls or unlabelled controls were included for each antibody and used to set the electronic gates on the flow cytometer. Cells were then incubated with Sytox Blue and analysed by FACS Canto II analyser (BD Biosciences). FACS data were analysed by FACSDiva software (BD Biosciences).

### Immunostaining

Frozen OCT sections or cultured cells were fixed in 4% paraformaldehyde for 10 min at RT, permeabilized with 0.2% Triton X-100 for 10 min and used for chromogen immunostaining or immunofluorescence.

For chromogen staining, sections were blocked with 0.3% hydrogen peroxide for 10 min followed by serum-free protein block (Dako) for 10 min. Sections were then incubated with CD49f primary antibody (1:100, clone GoH3; BD Pharmingen) or rat IgG2a isotype control (1:200; BD Pharmingen) overnight at 4°C, followed by biotinylated goat anti-rat secondary antibody (1:200; Vector Laboratories, Burlingame, CA, US) for 60 min at RT followed by streptavidin-horse radish peroxidase (1:200; GE Healthcare) for 60 min at RT and then 3,3’-diaminobenzidine (Sigma-Aldrich) for 5 min. Sections were then counterstained with haematoxylin, dehydrated and mounted with DPX. Sections were examined under Olympus BX41 microscope (Olympus), and images taken using Olympus DP25 digital camera.

For immunofluorescent staining, sections or cultured cells were blocked with Protein Block (Dako) and stained for CD271 in combination with either von Willebrand Factor (vWF) or alpha smooth muscle actin (αSMA). For CD271 and vWF, sections were incubated with a primary antibody against vWF (1:50; clone F8/86 mouse IgG_1κ_; Dako) for 1 hr at 37°C, followed by Alexa Fluor 488 conjugated donkey anti-mouse IgG (1:500; Abcam) for 30 min at RT, washed three times and incubated with 10 μg/ml mouse IgG to block any residual Alexa Fluor 488 anti-mouse IgG. Sections were then incubated with PE-conjugated CD271 (1:100, mouse IgG1; Miltenyi Biotec) overnight at 4°C. For CD271 and αSMA, sections were incubated with PE-conjugated CD271 (1:100) and rabbit anti-αSMA (1:100, Abcam ab5694) for 1hr at RT, washed then incubated with Alexa Fluor 568 anti-mouse IgG (1:500) and Alexa Fluor 488 conjugated donkey anti-rabbit IgG (1:500; Molecular Probes).

Fixed cultured cells were stained with either CD271 (1:100) or α-SMA (1:100) for 1hr at RT, washed twice with PBS and further incubated in donkey anti-mouse IgG Alexa Fluor 568 (1:500). Immunofluorescence preparations were counterstained with Hoechst 33528 (4 μg/ml; Molecular Probes), mounted with fluorescent mounting media (Dako) and examined using Nikon C1 confocal microscope (Nikon Instruments Inc).

### Statistical Analysis

All analyses were done with GraphPad PRISM software (version 6; GraphPad Software Inc., San Diego, CA, US). Data are shown as mean ± SEM. Two-way ANOVA and post hoc test (Tukey’s correction) were used for comparisons between groups. Results were considered statistically significant when the P value <0.05.

## Results

### Identification and characterisation of ovine endometrial MSC (eMSC)

The ovine endometrium differs from human in that it has aglandular caruncles and glandular intercaruncles (S1 Fig). We therefore first examined the cellular yield of single cell suspensions derived from caruncular and intercaruncular regions. The total cell numbers were 1.84 x 10^5^ ± 0.46 x 10^5^ cells/g tissue (n = 5) and 2.86 x 10^5^ ± 0.95 x 10^5^ cells/tissue (n = 4) respectively and was not significantly different (p> 0.05). Therefore for this study, cells were isolated from the whole ovine endometrium including from both caruncles and intercaruncles.

Initially, unfractionated cell suspensions containing both endometrial epithelial and stromal cells were seeded at cloning density (50 cells/cm^2^). In contrast to cloning or even standard culture of human endometrial cells, ovine epithelial cell clones outgrew the stromal clones (S2 Fig).

**Fig 1:**
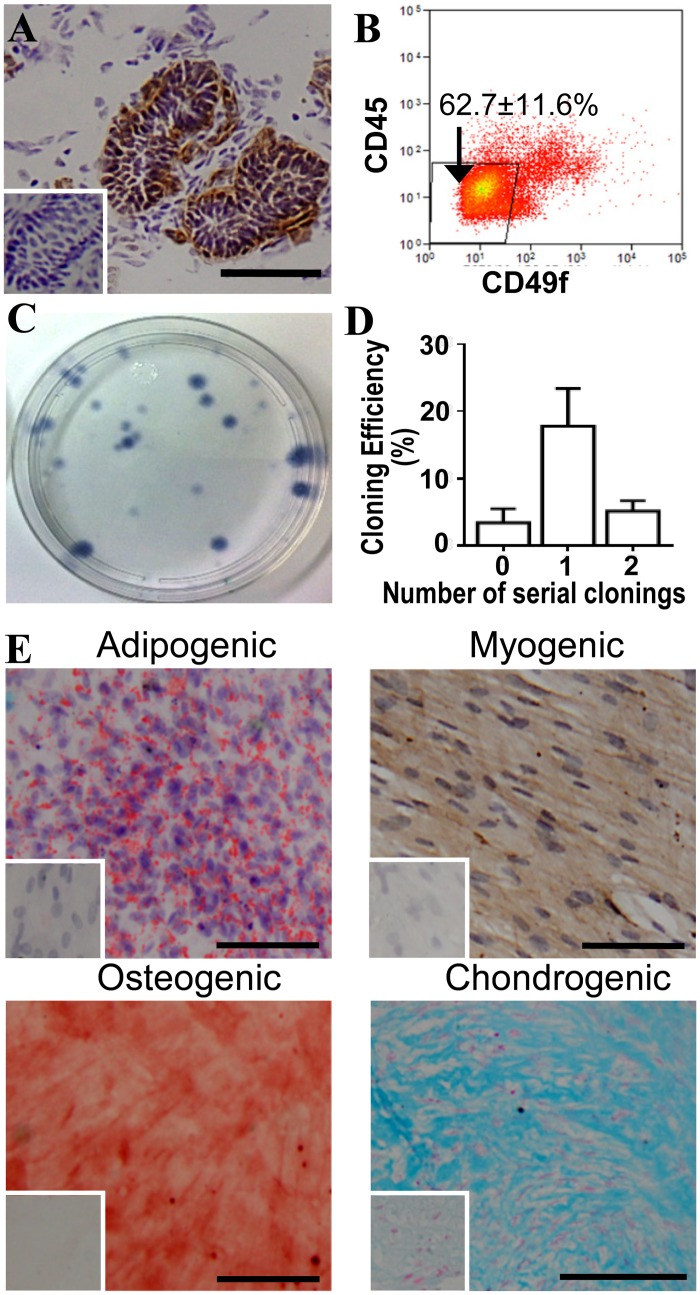
MSC properties of ovine endometrial stromal cells purified with CD49f antibodies to remove epithelial cells. (**A**) Immunohistochemical staining of ovine endometrium showing glandular epithelial cells positive for CD49f (brown). Inset, isotype control. (**B**) Representative flow cytometry dot plot for CD49f and CD45 (leukocyte marker) sorting ovine endometrial cell suspensions. The double negative population are the stromal cells, the percentage shown is mean ± SEM (n = 4). (**C**) Culture plate showing colony forming units from CD49f^-^CD45^-^ sorted endometrial stromal cells. (**D**) cloning efficiency (CE) of serially cloned CD49f^-^CD45^-^ endometrial stromal cells. Two to three large colonies from each ovine samples (n = 3) were serially cloned twice. (**E**) Differentiation of CD49f^-^CD45^-^ sorted stromal cells into mesodermal lineages after 4 weeks culture in induction media. Images are representative of n = 3 biological experiments. Insets, controls cultured in 1% serum DMEM media. Scale bar, 50 μm.

In order to isolate pure stromal cells from ovine endometrium we next determined a surface marker for the ovine epithelial cells to enable their removal by sorting using magnetic beads or a cell sorter. By immunohistochemistry, CD49f (α_6_ integrin) was expressed on glandular and luminal epithelial cells (Fig 1A). To quantify the stromal population by flow cytometry, we dual-labelled ovine endometrial cells with CD45 and CD49f antibodies to exclude leukocytes and epithelial cells, respectively. This CD49f^-^CD45^-^ stromal population comprised 62.7 ± 11.6% (n = 4) of the endometrial cell population (Fig 1B). CD49f^-^CD45^-^ stromal cells were clonogenic (Fig 1C) with a mean cloning efficiency of 3.48 ± 1.79% (n = 4) (Fig 1D). CD49f^-^CD45^-^ stromal cells also underwent self renewal *in vitro* as shown by their ability to serially clone at least twice (Fig 1D) with the first serially cloned cells having a cloning efficiency of 17.8 ± 5.6% (n = 3). CD49f^-^CD45^-^ stromal cells also underwent differentiation into multiple mesodermal lineages; adipogenic, myogenic, osteogenic and chondrogenic (Fig 1E).

**Fig 2:**
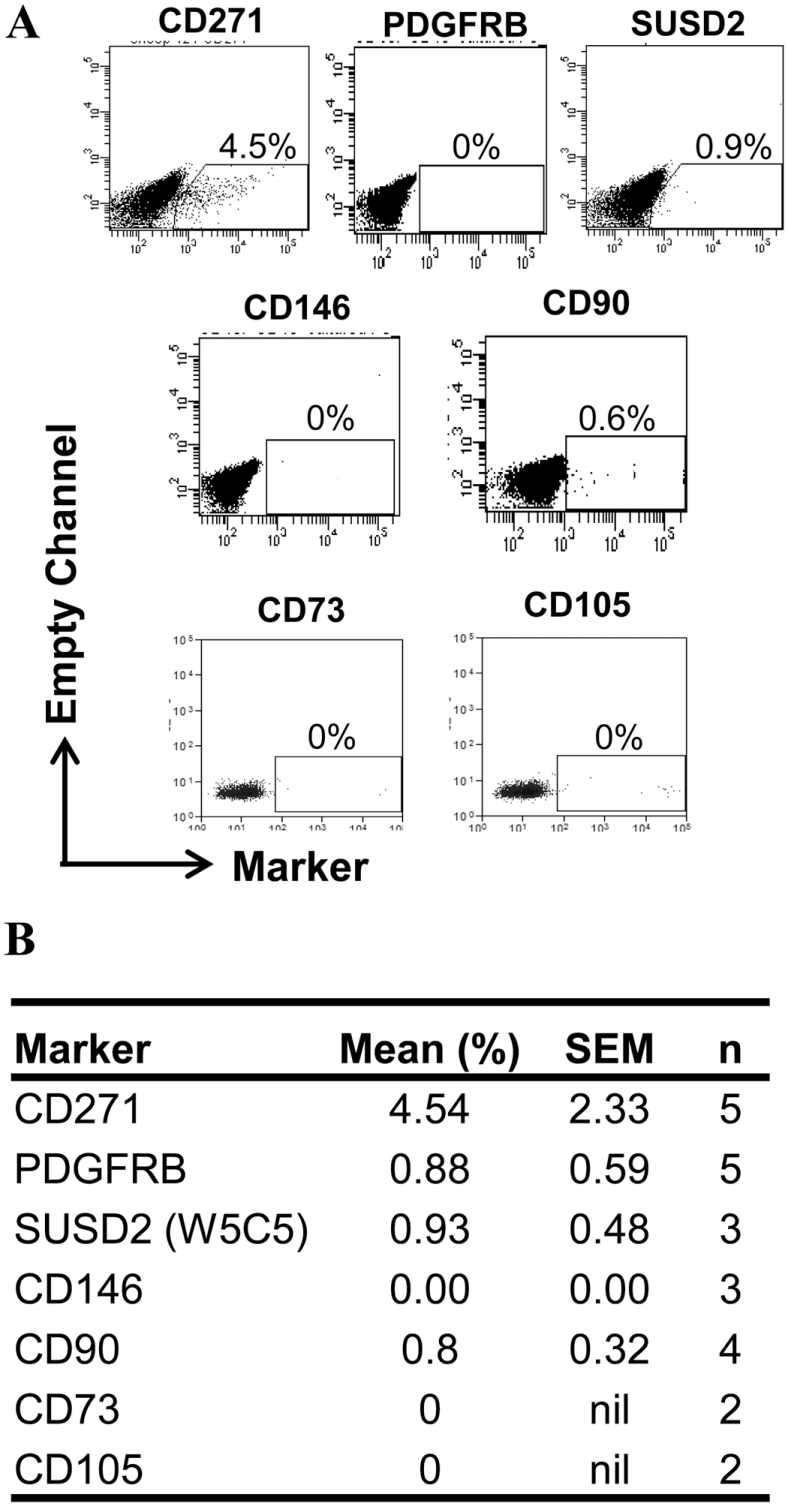
Surface phenotype of CD49f^-^CD45^-^ ovine endometrial stromal cells. (**A**) Representative flow cytometry plots for phenotypic data of CD49f^-^CD45^-^ cells with their relative percentages of positive cells and (**B**) summarised in the table.

Having identified an eMSC population in ovine endometrium, we next sought to determine if several key surface markers used to obtain purified populations of human bmMSC and eMSC were expressed on ovine CD49f^-^CD45^-^ stromal cells. However, we found none of the antibodies we had previously used for human eMSC purification, PDGFRB, SUSD2 (W5C5), CD146,or several phenotypic MSC markers, CD90, CD73 and CD105 (Fig 2) were detected, suggesting lack of cross reactivity between human and ovine species. Percentages below 1% were regarded as negative. Since CD271 has been reported on ovine bmMSC [24], we next examined CD49f^-^CD45^-^ ovine endometrial stromal cells for expression of CD271. Fig 2A and 2B shows that a small population of CD271^+^ cells were present in the purified endometrial stromal fraction.

### Prospective isolation of ovine eMSC

**Fig 3:**
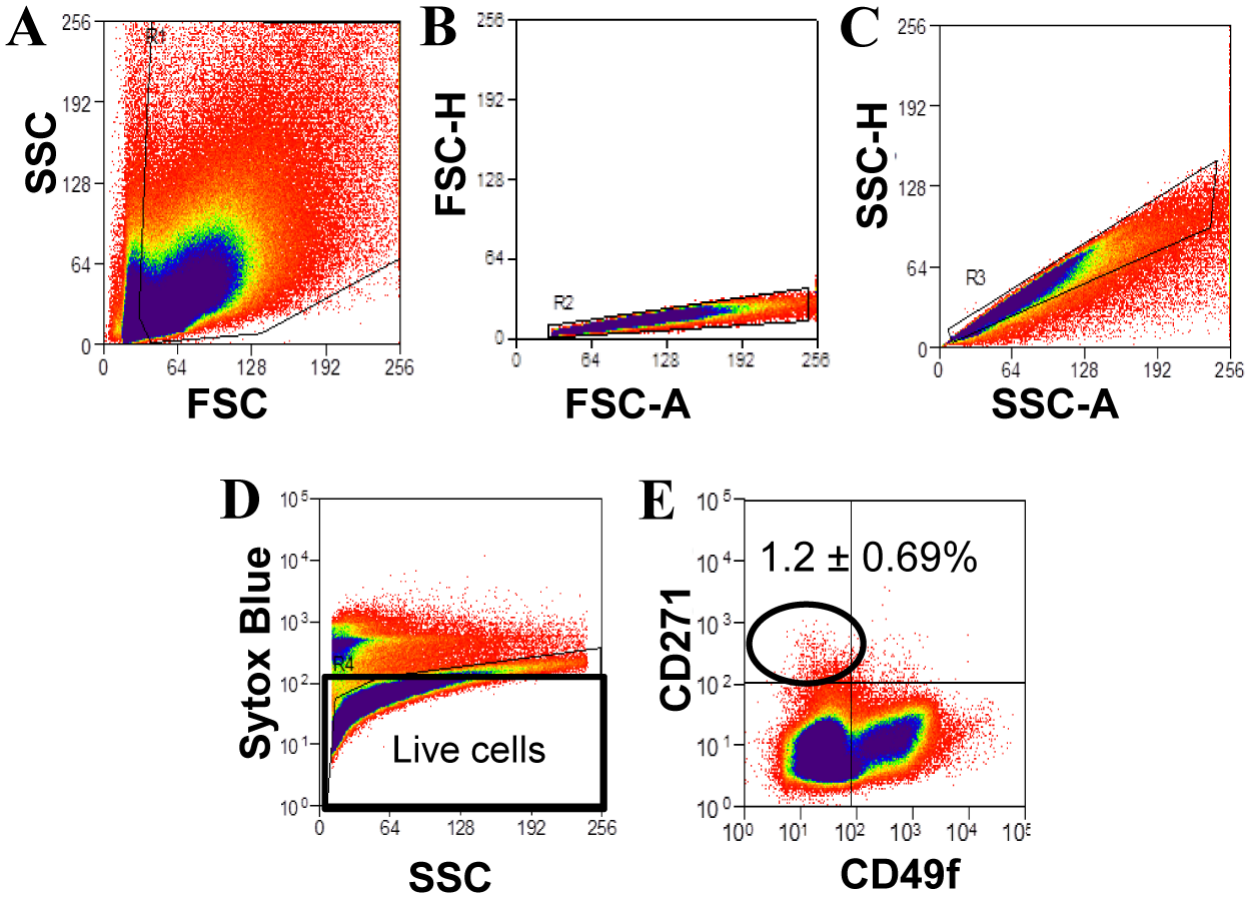
CD271 identifies a stromal subpopulation in ovine endometrium. (**A-E**) Flow cytometry cell sorting of freshly isolated ovine endometrial cells double labelled with CD271 and CD49f antibodies. (**A**) Scatter dot plots to set gates for viable cells and (**B-C**), for single cells, (**D**) dead cells were removed by Sytox Blue gating. (**E**) Representative dot plot for dual staining of stromal cells with CD271 and CD49f antibodies of n = 10 biological samples.

**Fig 4:**
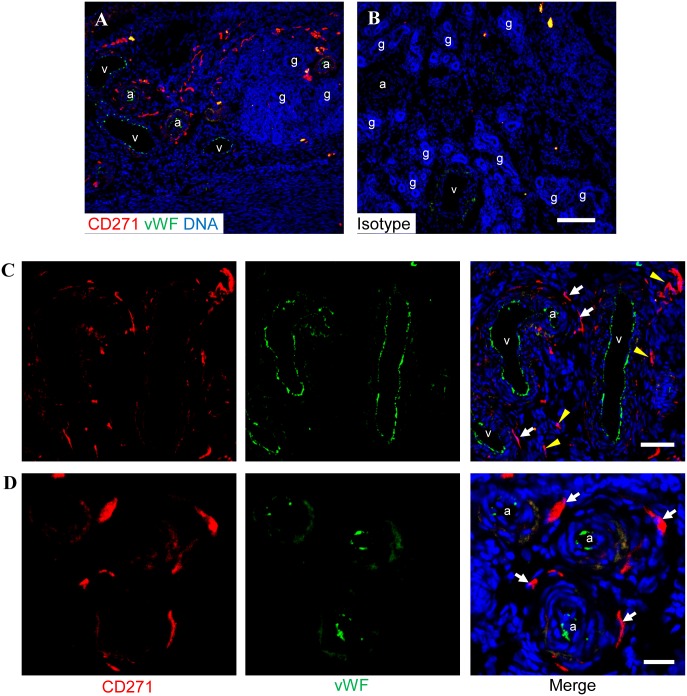
Immunofluorescence staining of ovine endometrium for CD271 and vonWillebrand Factor. **(A)** A low power micrograph of endometrium shows CD271 (red) relative to venules (v) and arterioles (a) that express vonWillebrand Factor (vWF, green). **(B)** Isotype control stained endometrium. **(C&D)** Some CD271^+^ cells were associated with vWF^+^ vasculature (white arrows) while others were not (yellow arrowheads). Nuclei are stained with Hoechst 33258 (blue). Scale bars: B = 100μm, C = 50μm, D = 20μm, A is at the same magnification as B. a, arteriole; g, gland; v; venule.

**Fig 5:**
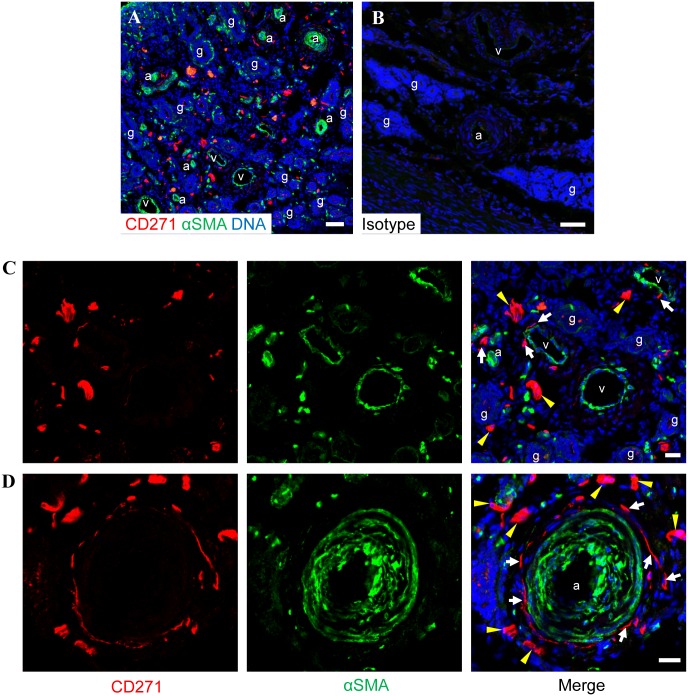
Immunofluorescence staining of ovine endometrium with CD271 and alpha-smooth muscle actin. (A) A low power micrograph of endometrium shows CD271(red) relative to cells expressing alpha-smooth muscle actin (αSMA, green) in the region surrounding venules (v), arterioles (a) and in the stroma between. (B) Isotype control stained endometrium. (C&D) Flattened CD271^+^ cells were associated with αSMA^+^ regions surrounding the vasculature (white arrows) but did not express αSMA, suggesting their location in the adventitia. CD271^+^ cells not closely associated with large vessels or arterioles (yellow arrowheads) also did not express αSMA. Nuclei are stained with Hoechst 33258 (blue). Scale bars: A&B = 50 μm, C&D = 20μm. a, arteriole; g, gland; v; venule.

Since there was a small CD271^+^ population in ovine endometrial stromal cell suspensions, the eMSC were extracted by double labelling with CD271 and CD49f antibodies and sorted as shown in Fig 3A–3E. Dual colour immunofluorescence frequently revealed CD271^+^ cells in the perivascular region surrounding vWF-expressing endothelial cells of arterioles and less frequently venules (Fig 4A–4D). Other CD271^+^ cell were not clearly associated with vWF^+^ vessels but were often arranged in a linear manner suggesting they may be associated with capillaries (Fig 4A, 4C and 4D). None of the CD271^+^ cells detected in the ovine endometrium expressed the pericyte marker αSMA (Fig 5A–5D) although some were closely associated with the αSMA^+^ region surrounding venules (Fig 5C) and arterioles (Fig 5D), suggesting their location in the adventitia of arterioles and venules.

### Cloning efficiency of ovine eMSC

**Fig 6:**
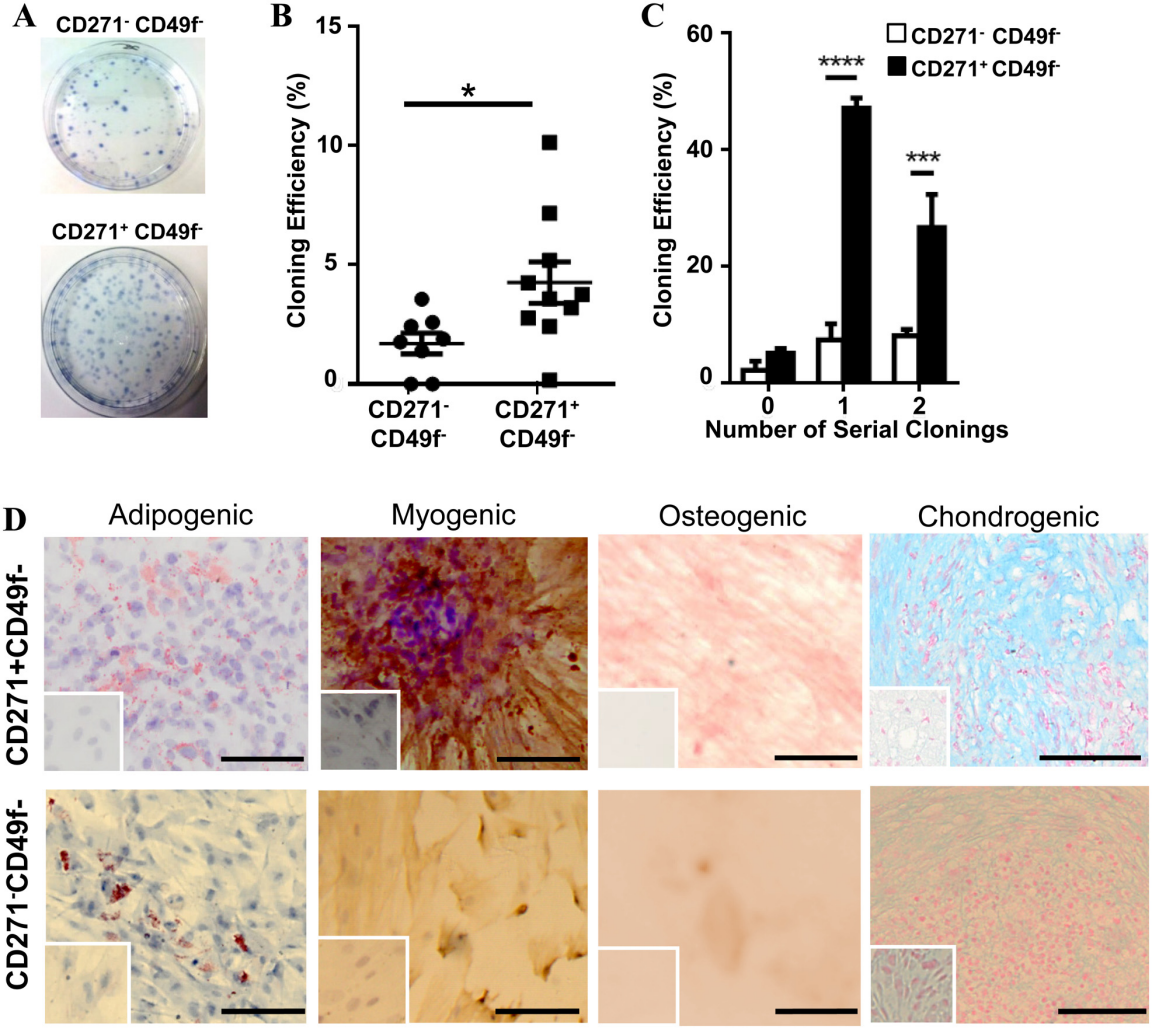
CD271^+^CD49f^-^ selects for ovine endometrial MSC. (**A**) Cloning plates showing representative examples from CD271^+^CD49f^-^ and CD271^-^CD49f^-^ stromal cells and (**B**) cloning efficiency of CD271^+^CD49f^-^cells (n = 10) and CD271^-^CD49f^-^ cells (n = 6). (**C**) Serial cloning of CD271^-^CD49f^-^ (white bars) endometrial stromal cells and CD271^+^CD49f^-^ (black bars) candidate eMSC. Two to three large colonies from each ovine sample (n = 3) were serially cloned 2 times. (**D**) CD271^+^CD49f^-^ and CD271^-^CD49f^-^ cells were induced to differentiate into mesodermal lineages by culturing in induction media for 4 weeks. Images are representative of n = 3 biological samples. Insets are controls cultured in 1% FBS DMEM media. * P<0.05, *** P<0.001, **** P<0.0001. Bars are mean ± SEM. Scale bar, 50 μm.

The cloning efficiency of CD271^+^CD49f^-^ and CD271^-^CD49f^-^ cells, as shown in Fig 6A–6C, was significantly higher in the CD271^+^CD49f^-^ cells (5.46 ± 1.14%, n = 10) compared with the CD271^-^CD49f^-^ population (1.58 ± 0.48%, n = 8) (p = 0.007). Serial cloning was further assessed for CD271^+^CD49f^-^ and CD271^-^CD49f^-^ cells, showing significantly higher cloning efficiencies at the first and second subcloning for the CD271^+^CD49f^-^ cells compared with CD271^-^CD49f^-^ cells (p< 0.0001 and 0.001 respectively) (Fig 6C).

### Differentiation of CD271^+^CD49f^-^ and CD271^-^CD49f^-^ cells

Multilineage differentiation was achieved by culturing the sorted CD271^+^CD49f^-^ and CD271^-^CD49f^-^ cells in specific culture induction media for four weeks. The CD271^+^CD49f^-^ cells differentiated into adipogenic, myogenic, osteogenic, and chondrogenic cells (Fig 6D, upper panel), staining strongly with Oil Red O, αSMA antibody, Alizarin Red and Alcian Blue (matrix) respectively, fulfilling another MSC criterion. The CD271^-^Cd49f^-^ cells showed weak differentiation into adipocyte, smooth muscle cell and chondrogenic lineages and failed to differentiate into the osteoblast lineage as there was no staining with Alizarin Red (Fig 6D lower panel). No differentiation was induced when cells were cultured in control medium.

### Characterising CD271^+^CD49f^-^cells

**Fig 7:**
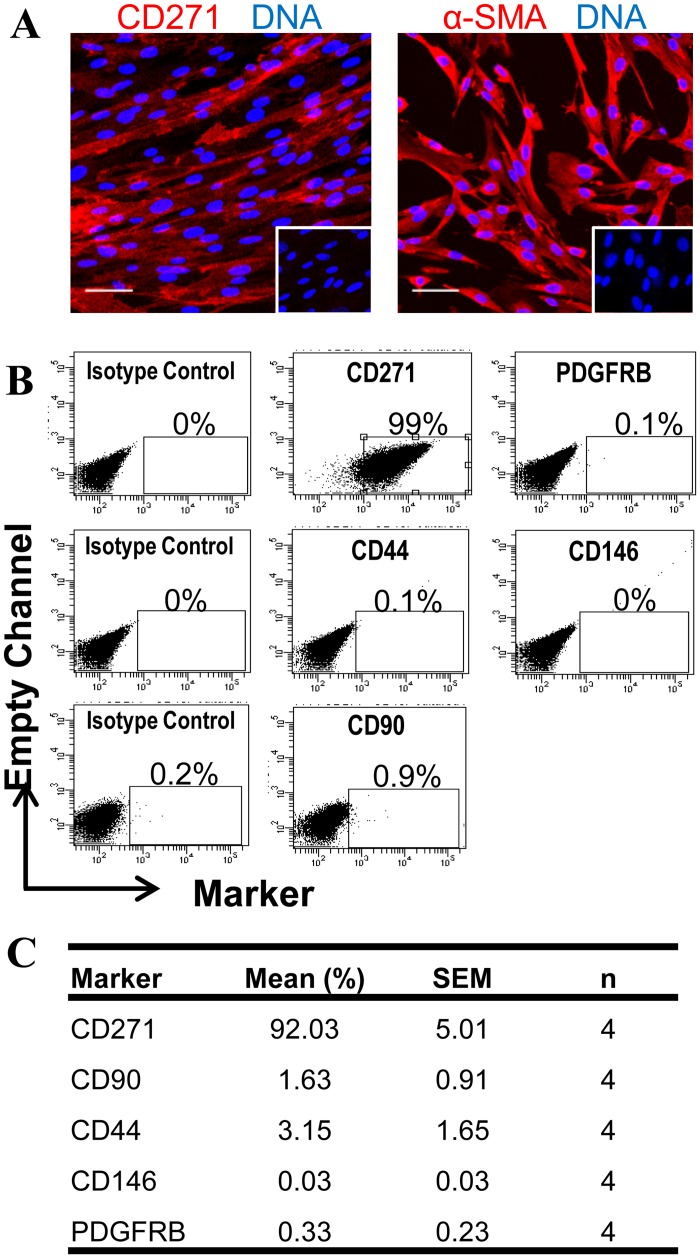
Phenotype of CD271^+^CD49f^-^ ovine endometrial cells by immunofluorescence and flow cytometric analysis. (**A**) CD271 and α-SMA immunostaining on passage 1 cultured ovine CD271^+^CD49f^-^ cells (red). Nuclei are stained with Hoechst 33258 (blue). Insets, Isotype controls. Scale bar, 50μm. (**B**) Representative flow cytometry dot plots for MSC markers representative of n = 4 biological samples and (**C**) table showing percent positive cells for each marker.

Fig 7 shows that 92.0 ± 5.0% of CD271^+^CD49f^-^ sorted cells still expressed CD271 after two to four passages in culture (n = 4) by flow cytometry and verified by immunofluorescence (Fig 7A). However continued passaging of CD271^+^CD19f^-^ results in their differentiation into CD271^-^ cells as the percentage CD271^+^ reduces to 51% at passage 5 (n = 2) and 40% at passage 6 (n = 2). A small number of CD271^-^CD49f^-^ cells become CD271^+^ during culture expansion; 15% and 12% at passages 5 and 6 respectively (data not shown). Cultured CD271^+^CD49f^-^ cells proliferate rapidly in culture with a population doubling time of 18.7 hr at passage 5 (n = 2) while CD271^-^CD49f^-^ cells had a slower rate of growth with a population doubling time at passage 5 of 30.0 hr (n = 2).

While CD271^+^ cells do not express αSMA *in vivo* (Fig 5), in culture they upregulate αSMA by passage 1 (Fig 7A). Other human MSC phenotypic markers were not expressed in cultured CD271^+^CD49f^-^ cells (Fig 7B and 7C), similar to ovine endometrial stromal cells (Fig 2). While CD271 proved to be effective in isolating eMSC from ovine endometrium, the human markers CD73, CD90, CD105, PDGFRB, or SUSD2 were not detectable on eMSC from the ovine endometrium (Fig 7B and 7C). A small percentage of CD271^+^CD49f^-^ cells (3.15 ± 1.65%, n = 4) expressed the MSC phenotypic marker CD44 (Fig 7), much lower than for human eMSC.

## Discussion

In this study we demonstrated for the first time that the ovine endometrium contains a small subpopulation of mesenchymal stem/stromal cells fulfilling several relevant MSC criteria; clonogenicity and multilineage mesodermal differentiation. We also demonstrated that CD271 is a selective marker enriching for the ovine eMSC subpopulation, as CD271^+^CD49f^-^ cells had a higher percentage of clonogenic cells, showed greater self-renewal *in vitro* and better ability to differentiate into mesodermal lineages compared to CD271^-^CD49f^-^ stromal cells. Similar to bmMSC and to human eMSC, the ovine eMSC were able to differentiate into adipogenic, myogenic, osteogenic and chondrogenic lineages.

Many of these CD271^+^ stromal cells were found in a perivascular location around arterioles and some venules in ovine endometrium, although they were not in close apposition to the endothelial cells as is the case for human eMSC. CD271^+^ cells were also located along presumptive vascular structures morphologically resembling capillaries. These presumptive capillaries did not immunostain with vWF antibody, which is more selective for endothelial cells of larger vessels [25]. Unfortunately other endothelial markers are unavailable for ovine endothelial cells. None of the CD271^+^ cells detected expressed αSMA suggesting that they are not pericytes. CD271^+^ cells surrounding arterioles may represent perivascular adventitial cells, a population previously reported to have MSC properties [26, 27].

CD271 has been previously used as a marker to isolate MSC from human bone marrow and umbilical cord blood [28]. Human umbilical cord CD271^+^ cells had slower proliferation rates than bone marrow CD271^+^ cells, although bone marrow CD271^+^ cells were more osteogenic than umbilical cord CD271^+^ cells [28]. Another comprehensive study examining the cross reactivity of the CD271 marker in multiple mammalian species found that CD271 enriched for clonogenic ovine, monkey, goat, canine, and porcine bmMSC concluding that it was a MSC marker for 6 large animal species useful for preclinical research [24]. Our study also identifies CD271 as a useful marker to purify ovine eMSC for our preclinical studies on their use in tissue engineering approaches for treating urological and gynecological disorders.

While all MSC have the accepted MSC properties, there are differences in some properties dependent on the tissue source of the MSC, particularly in their capacity to differentiate into the various lineages [29]. Cloning efficiencies for ovine eMSC and bmMSC are similar; 5.5% versus 4.6% [30]. The endometrium provides an additional valuable tissue from which MSC with high proliferative and broad differentiation capacity can be obtained.

The lack of specific MSC surface markers in tissues from non-human species has been described, i.e. for ovine bmMSC [30]. In line with the findings from this study, currently available human MSC phenotypic markers CD73, CD105, PDGFRβ, or CD146 were also not detectable on ovine bmMSC. In this study, we found that our selective markers for prospective isolation of human eMSC were either not expressed on ovine eMSC or the SUSD2 (W5C5), PDGFRB and CD146 antibodies did not cross react with the ovine epitopes. Neither were the antibodies to several of the phenotypic surface markers of bmMSC (CD73, CD90, CD105) immunoreactive with ovine endometrial stromal cells. The identification of CD271 as a specific marker of a small subpopulation of ovine endometrial stromal cells with eMSC properties allows the prospective isolation of ovine eMSC. Isolating pure eMSC is important to avoid a heterogeneous cell population in the stromal fraction [29], particularly for tissue engineering applications where cells with maximum proliferative potential are required.

It was not possible to culture ovine eMSC without first removing the epithelial population, which unlike human endometrial epithelial cells, possessed high proliferative capacity, outcompeting the stromal population. The sheep for this study were all parous and several years old. It has already been described previously that, unlike in humans, there was no correlation between age and bmMSC proliferative potential or age and MSC numbers in the ovine model [31].

The ease of access of endometrial tissue is a significant advantage of using this cell source from humans. Characterisation of ovine eMSC allows this source of MSC to be investigated in a large pre-clinical animal model. For example we are investigating their use as a cell based therapy for pelvic organ prolapse and the ovine model is ideal for testing this prior to clinical trials [32, 33]. eMSC were recently discovered in human endometrium [9, 10], where they likely have a role in endometrial regeneration each menstrual cycle. They are also believed to play a role in non-menstruating species and have been identified as label retaining cells in mice [34, 35]. More recently eMSC were also described in a larger animal, the pig [36], however the cloning efficiency was low in this species. This could be because the authors examined the unsorted stromal fraction. In non menstruating species it is suggested that eMSC have a role in endometrial remodelling that occurs each estrus cycle, and in particular in regenerating the stromal vascular component of the endometrium following delivery of the placenta and foetuses.

Tissue engineering has become popular during the last decade with increasing numbers of successful clinical applications, particularly in the urology and gynecology fields as exemplified in the reconstruction of the bladder and the vagina [37]. Due to similarities in anatomy to humans, sheep have been frequently used as a large preclinical animal model, for example the implantation of autologous bmMSC combined with ceramic to generate bone tissue [38]. The identification and availability of eMSC in the ovine uterus can facilitate further research in the gynecology and urology field using an autologous model. Tissue engineering attempts combining eMSC and novel materials for pelvic organ prolapse repair have provided promising data in a small animal model [39] and now need to be examined in a large preclinical animal model. The sheep is an excellent model for preclinical studies [40]. Both Gynecology and Urology disciplines are investigating novel treatment options using cell seeded scaffold devices as tissue engineering constructs for either fascia repair or the restoration of damaged and under developed organs. The availability of autologous eMSC in a large animal model will facilitate future research in this field, having applications for very common conditions such as pelvic organ prolapse and stress urinary incontinence.

## Conclusion

Ovine endometrium contains a small population of mesenchymal stem/stromal cells which are enriched in the CD271^+^ CD49f^-^CD45^-^ subpopulation.

## Supporting Information

S1 Fig H&E staining of ovine uterus. Dotted line delineates the endometrium and myometrium. C, caruncle; IC intercaruncle. Scale bar, 200 μm.
(PDF)

S2 Fig Ovine endometrial epithelial contamination on stromal cell cloning plate. The well was seeded with 50 unsorted ovine endometrial stromal cells/cm^2^ and cultured for 14 days. Contaminating epithelial clones (dotted) competed with and overgrew stromal clones (square) on the cloning plate.
(PDF)
