# Supplementary figures and images for: Isolation and Characterisation of Mesenchymal Stem/Stromal Cells in the Ovine Endometrium
Source: PLoS One. 2015 May 18;10(5):e0127531. doi: 10.1371/journal.pone.0127531 (PMC4436363; doi:10.1371/journal.pone.0127531)

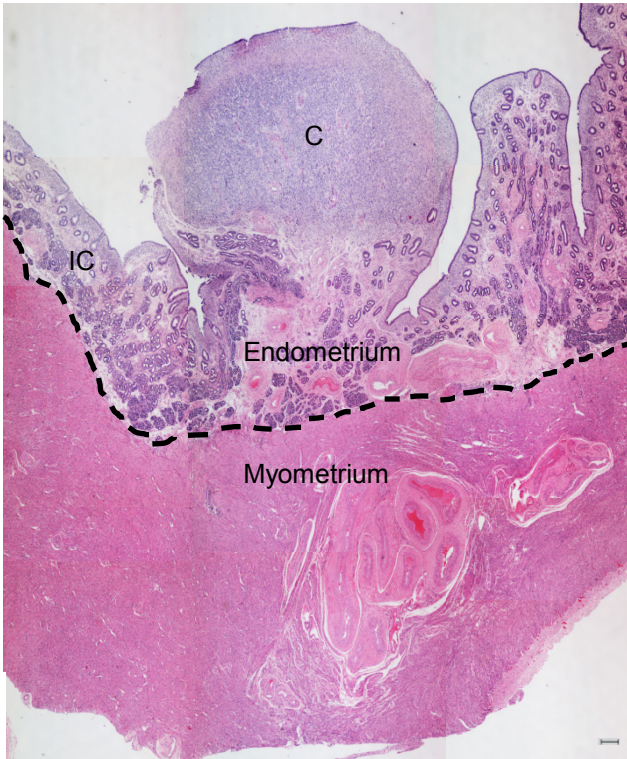

Supplement: S1 Fig — Dotted line delineates the endometrium and myometrium. C, caruncle; IC intercaruncle. Scale bar, 200 μm. (PDF) [file pone.0127531.s001.pdf]

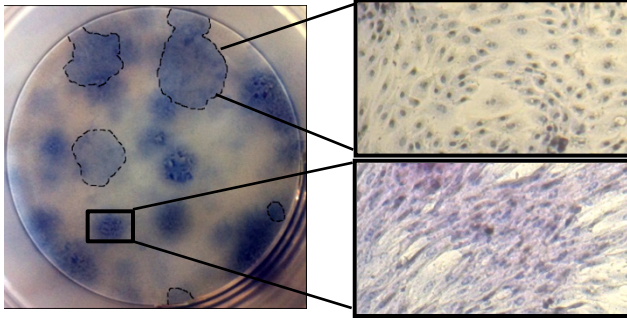

Supplement: S2 Fig — The well was seeded with 50 unsorted ovine endometrial stromal cells/cm2 and cultured for 14 days. Contaminating epithelial clones (dotted) competed with and overgrew stromal clones (square) on the cloning plate. (PDF) [file pone.0127531.s002.pdf]
